# Bi‐peaked R wave in HRV: A technical pitfall or ventricular pathology

**DOI:** 10.1002/joa3.70111

**Published:** 2025-06-13

**Authors:** Anish Singhal, Billa Anala, Madhuri Taranikanti, Nitin Ashok John, Naveen Ravi

**Affiliations:** ^1^ Department of Physiology AIIMS, Bibinagar Hyderabad India

**Keywords:** bi‐peaked R wave, double R wave, ECG, heart rate variability

## Abstract

During routine heart rate variability recording in an elderly male, bi‐peaked R waves were recorded consistently. Initially, it was thought to be because of technical issues, which were ruled out systematically; next, it was believed to have been caused by cardiac pathology, which was evaluated using ECG and echocardiography.

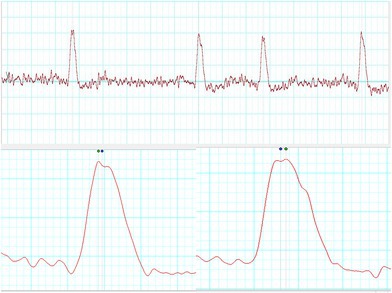

1

An 80‐year‐old moderately built and nourished elderly male patient with complaints of on and off dizziness, postural instability, and gait disturbances was examined. He had negative history of hypertension, diabetes mellitus, cardiac illnesses, or any other chronic illnesses. He was a nonalcoholic and nonsmoker. He has no history of consumption of medications over a prolonged duration of time. On examination, his pulse rate was 90 beats per minute, SPO_2_ was 98% on room air, respiratory rate of 15 breaths per minute and his blood pressure (BP) was 130/80 mm of Hg. He had no signs of pallor, icterus, cyanosis, edema, clubbing, or lymphadenopathy. CNS examination was significant for motor system abnormalities with the presence of both brady and hypo kinesia, mild rigidity in all limbs, and tremors in both hands. His cranial nerve examination was within normal limits. Other systemic examination revealed a normal cardiovascular system, respiratory system, and per abdomen. For dizziness, postural instability he was instructed to record his BP at home on a regular basis for a week and come back for follow up. The findings (Table 1) showed wide diurnal and intraday fluctuations in BP, varying from 120/63 to 188/97 mm Hg.

**TABLE 1:** The patients home‐based BP recording over a duration of 9 days.

Day	Time of day	SBP/DBP in mm Hg	Pulse rate (beats per minute)
1	Morning	188/97	76
	Night	163/85	72
2	Morning	120/63	50
	Afternoon	151/92	59
	Night	171/95	65
3	Morning	111/71	47
	Night	124/66	43
4	Morning	139/71	51
	Afternoon	151/84	62
5	Morning	119/72	59
	Afternoon	162/91	71
	Night	126/64	49
6	Morning	153/85	73
	Night	131/69	64
7	Morning	117/62	47
	Night	123/67	48
8	Morning	130/69	50
	Night	121/71	42
9	Morning	113/77	78

The patient was then referred to Department of Physiology for autonomic evaluation in view of his symptomatology. He underwent autonomic function test (AFT) and heart rate variability (HRV) analysis. The data acquisition was done by AD Instruments PowerLab and data analysis was done using the LabChart 8 Pro software.

The recording and interpretation of HRV in our laboratory follows established protocols.^1^, ^2^, ^3^

During AFT, his BP in supine positing measured in his right arm was 128/80 mm of Hg, which fell to 90/60 mm of Hg immediately after standing. The fall in SBP was >30 mm of Hg, whereas DBP fell by >20 mm of Hg, suggesting severe orthostatic hypotension. The change in Diastolic BP in Isometric exercise (Handgrip test) also revealed a fall of >5 mm Hg. Other tests including the E:I ratio in deep breathing test and 30:15 ratio of lying to standing were also low. All the above results suggest a severe loss of sympathetic and parasympathetic reactivity.

For HRV, we collect time and frequency domain parameters. The time domain parameters include mean HR, SDNN (Standard deviation of normal‐to‐normal intervals), RMSSD (root mean square of successive differences), and pNN50 (percentage of successive normal‐to‐normal intervals that differ by more than 50 milliseconds).

Frequency domain parameters include TP (Total Power) LF (low frequency) power, HF (high frequency) power, and LF/HF ratio. Total power (TP) represents overall variability.

HF reflects parasympathetic activity, LF can indicate sympathetic activity, and the LF/HF ratio is a measure of sympathovagal balance.^4^

The patient's HRV also revealed a severe loss of autonomic tone at rest. During his HRV recording, the R waves on electrocardiogram (ECG) were found to be bi‐peaked, as shown in Figures 1, 2, 3 (zoomed in Figures 2 and 3). Thus, it was analyzed by both the HRV (Blue dot) and ECG (Green dot) modules available in the LabChart 8 Pro software at a sampling rate of 1000 Hz and low pass filtering of 50 Hz.

**FIGURE 1:**
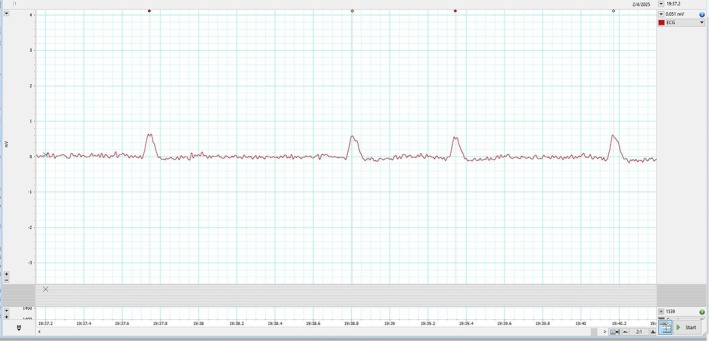
HRV recordings in the patient.

**FIGURE 2:**
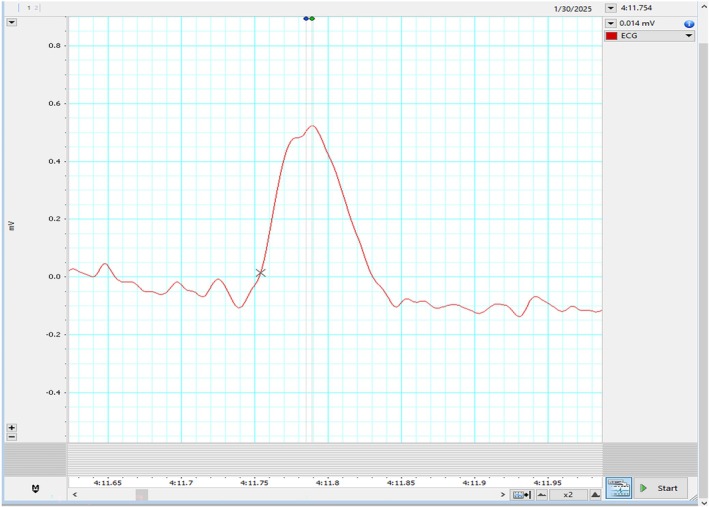
Zoomed in image of the bi‐peaked R waves that were seen during HRV recording in the patient.

**FIGURE 3:**
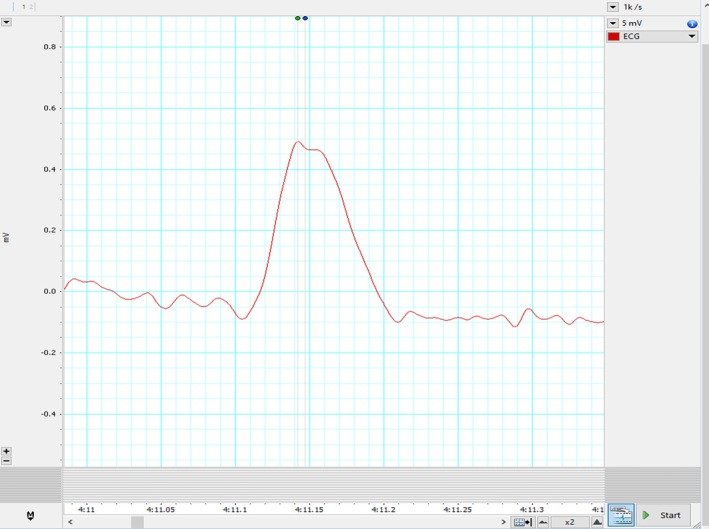
Another set of zoomed‐in images showing bi‐peaked R waves that were seen during HRV recording in the patient.

Represented below is another kind of bi‐peaked R waves that were seen during HRV recording in the patient Figure 3. This also got analyzed by both the HRV (Blue dot) and ECG (Green dot) modules available in the LabChart 8 Pro software. The interesting aspect to note is, in contrast to Figure 2, here the amplitude analyzed by the HRV module (Blue dot) is later compared to the peak analyzed by the ECG module (Green dot).

On reviewing the probable causes of bi‐peaked R wave, they can be

1. Physiological causes like patient movement including those of respiratory movements^5^, ^6^;
2. Technical/Instrumentation Causes because of poor skin preparation, electrode issues or signal processing artifacts^5^, ^6^ or
3. Cardiac Causes including ventricular hypertrophy, Wolff–Parkinson–White Syndrome, causes for premature beats or bundle branch block^7^

Thus, first to rule out technical/iatrogenic causes of bi‐peaked R wave, the recording was temporarily stopped. The proper anatomical placement of all the electrodes was checked, any loose electrodes were reapplied, skin was again prepared, and software's sensor detection was re‐confirmed. To avoid any movement artifact, instructions were repeated to the patient and were confirmed by physical inspection. The recording was restarted, but the bi‐peaked R waves still persisted, ruling out the first two causes of bi‐peaked R waves.

To understand the discrepancy of analysis modules, the reasonable reason for these findings (Figures 2 and 3) seems to be the difference in fine‐tuning of the waveform (peak) detection algorithms used by the HRV and ECG analysis modules. The ECG module emphasizes more on the wave characteristics, whereas the HRV module primarily focuses on the RR interval. This caused a lag in peak detection on the same data. Though this explanation also does not seem to have clarified the cause of double peak, but this information is necessary to understand software's used for HRV analysis.

To rule out the third, that is, pathological causes, the patient was referred for further cardiac evaluation, which included a 12 lead ECG and 2D Echocardiography. His ECG was recorded by routine clinical ECG machine as is shown in Figure 4, and revealed mostly normal findings but for mild prolongation of QRS complex. However, one should know that the routine clinical ECG machine might have a lower sampling rate and higher softening of waves, which might have smoothened the split R wave and represented as normal ECG, despite transient changes. A typical ECG machine generally uses a sampling rate of 250–500 Hz, with high‐ and low‐pass filters set at 0.05 Hz or 0.5 Hz and 100 Hz or 150 Hz, respectively.^8^

**FIGURE 4:**
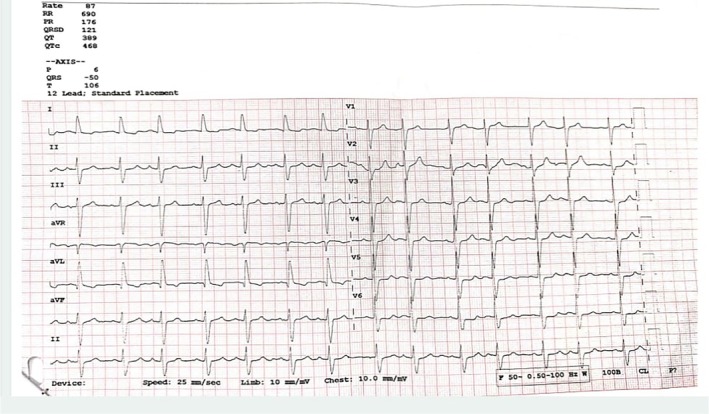
ECG recoding of the patient.

Echocardiography showed mild concentric left ventricular abnormalities with grade 1 diastolic dysfunction. There were no regional wall motion abnormalities also. The ECG and ECHO thus helped us to rule out a cardiac pathology behind the bi‐peaked R waves.

Physiologists should be aware of the findings of the bi‐peaked R waves during the recording of HRV, as knowledge about them can prevent misinterpretation and misdiagnosis. Additionally, the simple flowchart (Figure 5) that has been mentioned as followed by us can be adapted in Autonomic labs as a part of their protocols and requirements.

**FIGURE 5:**
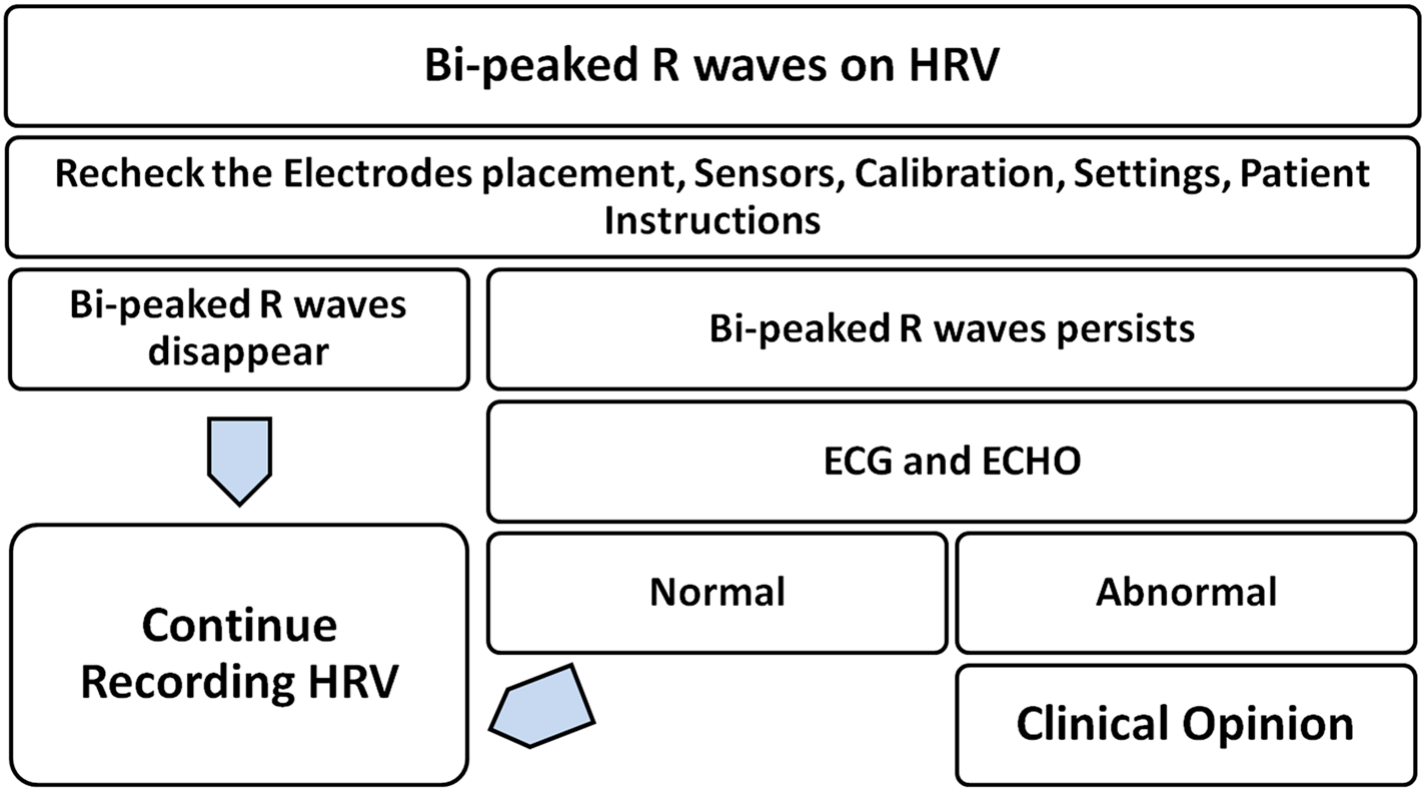
A flowchart showing the series of steps to be taken when the bi‐peaked R waves are encountered on HRV.

However, it is not clear why the bi‐peaked R waves were seen in our case. But it may be because of transient conduction slowing because of autonomic/pathologic modulations affecting the conduction velocity or some unknown filtering artifacts mimicking split R that was detected in the HRV assessment equipment but missed by the routine clinical ECG machine.

Awareness about the bi‐peaked R waves and the possible causes for them is important among physiologists, clinicians, and technicians. As these can appear abruptly and randomly, they can cause confusion. In such instances, interpretation of ECG, ECHO, and ruling out technical glitches is imperative. As described, incorporation of the above protocol can help during the course of HRV recordings.

More information and case studies are required for further validation of the protocol.

## FUNDING INFORMATION

None of the authors received any financial aid for this study.

## CONFLICT OF INTEREST STATEMENT

Authors declare no conflict of interests for this article.

## CONSENT

Informed consent from the patient was obtained for this study.

## Data Availability

Data available on request from the authors.
